# A mechanistic framework linking the oral microbiome to Alzheimer's disease through neuroinflammation

**DOI:** 10.1177/13872877261456324

**Published:** 2026-06-12

**Authors:** Manon J. A. P. Evers, Bastiaan P. Krom, Caroline A. de Jongh

**Affiliations:** 1 Department of Preventive Dentistry, 1192 Academic Centre for Dentistry Amsterdam (ACTA), University of Amsterdam and Vrije Universiteit Amsterdam, Amsterdam, the Netherlands; 2 Department of Oral Biochemistry, 1192 Academic Centre for Dentistry Amsterdam (ACTA), University of Amsterdam and Vrije Universiteit Amsterdam, Amsterdam, the Netherlands; 3 Department of Oral Cell Biology, 1192 Academic Centre for Dentistry Amsterdam (ACTA), University of Amsterdam and Vrije Universiteit Amsterdam, Amsterdam, the Netherlands; 4 Department of Periodontology, 1192 Academic Centre for Dentistry Amsterdam (ACTA), University of Amsterdam and Vrije Universiteit Amsterdam, Amsterdam, the Netherlands

**Keywords:** Alzheimer's disease, bacteria/microbiome/microbiota, microglia, neuroinflammation, periodontal disease

## Abstract

Alzheimer's disease (AD) is a growing problem in our society and the most common form of dementia. This neurodegenerative disease is characterized by neuroinflammation and the accumulation of amyloid-β (Aβ) and tau. Previous studies have found associations between the oral microbiome and AD. This review aims to elucidate the role of the oral microbiome in AD, through neuroinflammation, and reviews the relationship between AD and bacteria and fungi. Studies have found bacteria (e.g., *Porphyromonas gingivalis*) and fungi (e.g., *Candida albicans*) in postmortem AD brains. Moreover, mice models have shown that oral microbes are able to cross the blood-brain barrier (BBB), and were correlated with activated microglia, neuroinflammation, and Aβ load. This review introduces a mechanistic framework that describes how oral microbes cause an inflammatory response resulting in AD pathology. Specifically, oral dysbiosis causes oral pathogens to disseminate into the bloodstream, this triggers an inflammatory response, subsequently activating microglia, ultimately resulting in AD pathology. This process can follow two pathways: First, there is a direct response of the immune system in the brain to oral pathogens that migrate through the bloodstream and cross the BBB, which causes neuroinflammation and activates microglia, leading to AD pathology. Second, an early-life systemic inflammation causes microglia to get into a “hyperactive” state, in which they respond in an exaggerated way to normal stimuli triggering immune responses throughout a person's life that result in AD pathology. This mechanistic framework provides new line of thought for future research on the question of causality of AD.

## Introduction

Alzheimer's disease (AD) is a growing problem in our society and the most common form of dementia. At this moment, the prevalence is estimated at 55 million people globally.^1^ This number is expected to grow to 152 million people in 2050.^2^ AD is a burdening disease for patients themselves, and their caregivers, but also for our society. Global reports from 2020 have estimated the total costs for the disease, including treatment and care, to amount to $305 billion.^3^ Despite the high prevalence of AD, which is higher in women than in men, and the extensive research that has been conducted, there are still many unanswered questions and therapy strategies have been unsuccessful thus far.^4^ In the past years, studies have been exploring the connection between the oral microbiome and AD, however this connection is not fully understood.

**Figure 1.**
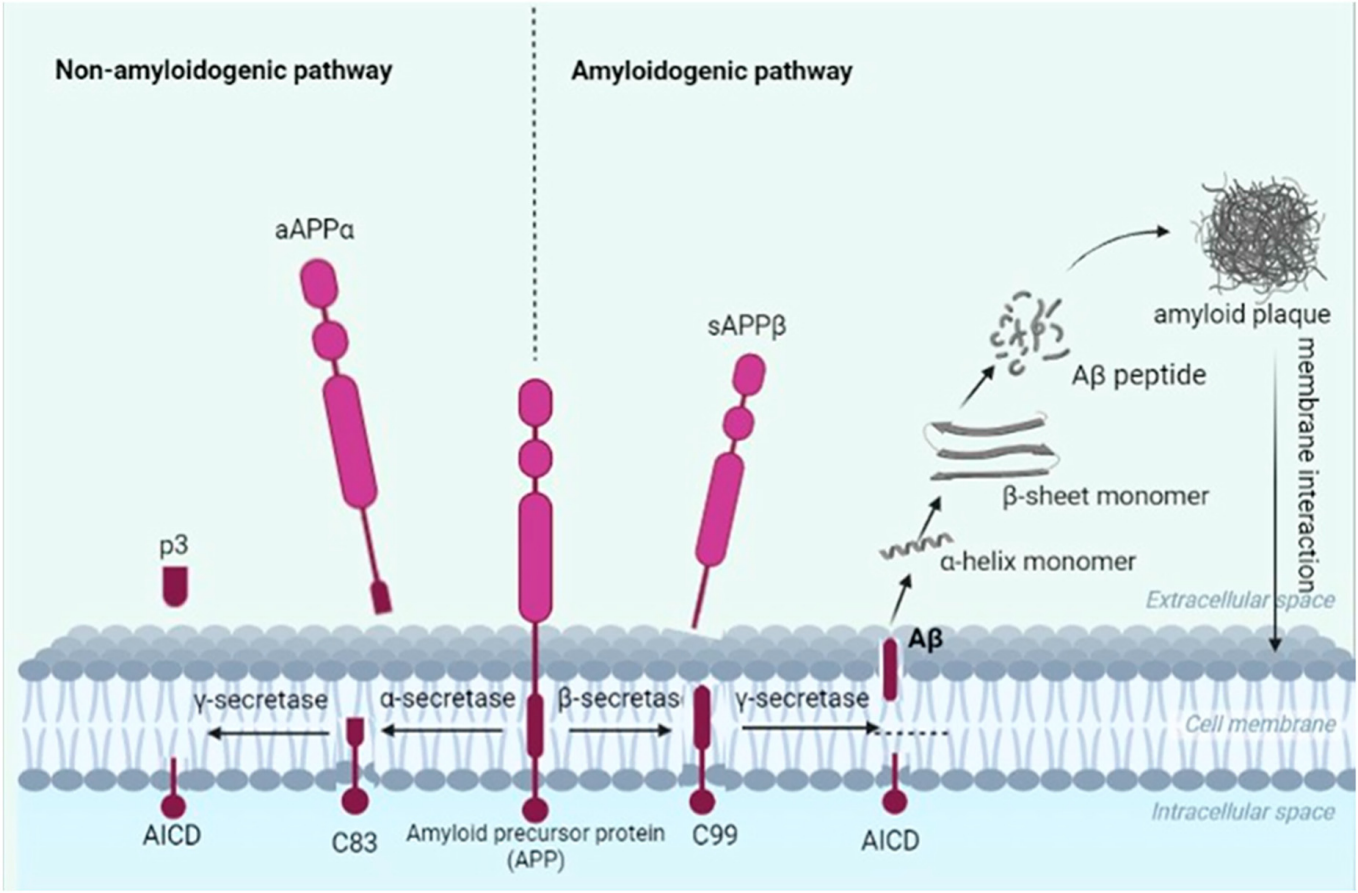
The cleavage of amyloid-β protein precursor (AβPP) in the non-amyloidogenic pathway and amyloidogenic pathway. In the non-amyloidogenic pathway, AβPP is cleaved by α-secretase and γ-secretase. In the amyloidogenic pathway, AβPP is cleaved by β-secretase and γ-secretase, leading to the formation of Aβ peptides, which will accumulate and consequently form Aβ plaques (Figure taken from Wojtunik-Kulesza and colleagues).^13^

AD is a progressive neurodegenerative disease and age is the biggest risk factor.^5^ The disease is characterized by many symptoms, including memory loss, cognitive decline, and language deficits.^6^ It is known that pathological processes of AD already start decades before the disease becomes symptomatic, making diagnosis and treatment difficult.^7^ The pathological hallmarks of AD include intracellular neurofibrillary tangles, composed of hyperphosphorylated tau, a microtubule-associating protein, and extracellular amyloid-β (Aβ) plaques (Figure 1).^8–10^ In the last years, the role of neuroinflammation in AD pathogenesis has been acknowledged. Studies have found activated microglia, the immune cells of the brain, co-localized with Aβ and tau. It has been hypothesized that pro-inflammatory microglia are directly linked to Aβ accumulation.^11,12^

The oral microbiome can be defined as “all microbes that exist in the oral cavity and their collective genome” and is the second largest microbiome of the human body.^14,15^ It consists of a diverse collection of 770 identified species, including Gram-positive, Gram-negative, aerobic, facultative anaerobic and strict anaerobic bacteria. In addition, fungi, viruses, and bacteriophages as well as protozoa are part of the oral microbiome in healthy oral cavities.^15^ The oral cavity is extremely dynamic and influenced by many different environmental factors.^15,16^ There is much variation between individuals, but also within individuals. This microbial diversity is, at least in part, related to niche diversity in the oral cavity. Ranging from the hard surfaces of the teeth, to keratinized hard mucosal surfaces and soft squamous mucosal surfaces, the oral cavity offers colonization sites for this range of organisms.^15^ Dysbiosis, which can be explained as a sudden shift in resident microbiota in the oral cavity, can result in an imbalanced microbial composition. This gives disease-promoting microorganisms the opportunity to grow and possibly cause disease and systemic inflammation.^17,18^

In the last years, studies have focused on the link between the oral microbiome and AD. Many studies have found correlations between these. Liu and colleagues found the salivary microbiome to be significantly less diverse in AD patients.^19^ Beydoun and colleagues showed that the combination of periodontal bacteria are correlated with the incidence and mortality of AD.^20^ Moreover, many studies have explored the association between AD and periodontitis, an inflammatory-infectious condition that damages the periodontium.^18^ Several studies have found correlations between periodontitis and AD.^21–26^ These studies suggest that the oral microbiome plays a role in the progression and/or onset of AD. However, thus far there are no studies providing evidence for the causal relationship between the oral microbiome and AD.

This review aims to elucidate the role of the oral microbiome in the development of AD, through neuroinflammation. This review explores the association between AD and different oral microbes, including bacteria and fungi, and introduces a mechanistic framework linking the oral microbiome to AD through neuroinflammation.

## Section I: Inflammation in Alzheimer's disease

It has been long thought that the accumulation of Aβ plays the central role in the pathogenesis of AD, known as the “amyloid-beta cascade” hypothesis.^27^ However, it has become clear that this hypothesis cannot fully describe the pathogenic mechanisms of AD, leading to more researchers rejecting this hypothesis and searching for other factors that play a role in the progression of this complex disease.^28–30^

It has become clear that neuroinflammation is at least one factor that plays an important role in the onset and progression of AD.^30^ This section describes the role of neuroinflammation in AD, highlights the role of microglia, and explores which role systemic inflammation possibly plays.

### The role of neuroinflammation in Alzheimer's disease

Neuroinflammation plays an important role in the pathogenesis of AD. Initially, an inflammatory response has a protective role in our body and promotes tissue homeostasis.^31^ However, a prolonged and excessive inflammatory response can become chronic, and cause neuronal damage and disease.^32^ In AD, chronic inflammation which cannot resolve by itself, results in inefficient clearance of misfolded proteins, like tau, contributing to protein aggregation, and thus the progression of AD.^31^ During inflammatory responses, there is a release of pro-inflammatory cytokines, including tumor necrosis factor, interleukin-1β (IL-1β), IL-6, and IL-8, that can result in impaired neurogenesis, synaptic dysfunction, and even neuronal cell death.^32,33^ Studies have shown increased levels of these cytokines in AD brains.^34–36^ Moreover, chemokines, nitric oxide, and reactive oxygen species are produced, which are all involved in the process of inflammation.^37^ Thus, this suggests that the chronic neuroinflammatory response observed in AD contributes to neuronal cell death and the progression of the disease.

### The role of microglia

Microglia are phagocytic immune cells of the central nervous system and make up for 10–15% of all cells in the brain.^9^ Microglia have many different functions, including neurogenesis, neuronal plasticity, and phagocytosis and clearance of toxic products.^9,38^ Moreover, they play a crucial role in the first line defense mechanism of the brain and can act as antigen-presenting cells.^39^ As studies have shown microglia to produce the highest levels of pro-inflammatory cytokines, as opposed to other glia cells, these brain cells are considered the primary cause of inflammation.^30^

Studies have shown microglia to be correlated to Aβ plaques, tau tangles, and memory loss.^40^ Postmortem case studies have found a co-localization of reactive microglia with Aβ plaques in AD brains.^11,12^ Moreover, *in vivo* and *in vitro* studies have found transcriptional responses in microglia to Aβ.^41^ It is thought that pro-inflammatory microglia are directly linked to the accumulation of Aβ. Microglia become less phagocytic once activated, resulting in poor phagocytosis of Aβ deposits.^42^ This would suggest that if these “hyperactive” microglia would become less active, the accumulation of Aβ would decrease. However, insufficient microglial activation could also result in neurodegeneration, suggesting that balance is key in the disease pathogenesis.^43^

Microglia scavenge around the brain and act as surveillance patrol. In the absence of foreign stimuli, they are not activated and called M0. Once activated, they proliferate, and change morphologically.^44,45^ Based on the stimulus they encounter, microglia can display two different activated phenotypes: M1, the pro-inflammatory phenotype; and M2, the anti-inflammatory phenotype. It is thought that in early stages of AD, microglia have a more anti-inflammatory phenotype (M2), which is associated with enhanced phagocytosis with the goal to clear protein accumulations and repair neuronal damage.^46,47^ However, when inflammation becomes chronic in further disease stages, it is thought that microglia switch to the pro-inflammatory phenotype, resulting in releasing higher levels of pro-inflammatory cytokines.^48^ However, recently a discussion has arisen regarding M1/M2 phenotypes, and whether microglia can be divided into just two phenotypes, or we should approach these phenotypes more as a spectrum.^49^ Interestingly, Gerrits and colleagues have shown distinct microglia profiles based on AD pathology.^50^ They performed snRNA sequencing on human AD brains, with both Aβ and tau pathology, or only Aβ pathology, and nondemented control brains. Their results show two different microglia populations: AD1-microglia, showing a phagocytic/activated phenotype similar to M2, which were linked to Aβ plaques; and AD2-microglia, that have not been identified before, which were linked tau pathology. These results were only found in microglia, not in other brain cells. This study provides new knowledge of the complex, but crucial role microglia play in the pathogenesis of AD.

### The role of systemic inflammation in Alzheimer's disease

Although, many studies have explored the working mechanisms of neuroinflammation in AD, these cannot yet be fully explained. The contradictory findings of these studies consequently raise an important question: “what is the initial starting point of AD pathology?”. Some studies suggest it starts with Aβ accumulation, and once the disease progresses further, this accumulation results in microglia activation and consequently inflammation.^9^ Recently, Pascoal and colleagues found that Aβ increases microglial activation, which influences the spread of tau across the cortex. They suggest that there is a close interaction between these three hallmarks and cooperatively determine dementia.^51^ Other studies propose that neuroinflammation initiates AD pathology, possibly long before the disease becomes symptomatic.^37^ There is increasing evidence suggesting that systemic inflammation, acute (e.g., infection) or chronic (e.g., autoimmune diseases), is correlated with AD.^52^ Studies have shown infections early in life or chronic infections, including diabetes and periodontitis, increase the risk for development of AD later in life.^53^ Moreover, the morphology of microglia appears to be similar in patients who have experienced systemic inflammation early in life and elderly with dementia.^37^ This systemic inflammation can result in neuroinflammation.^52^ Possibly, the initial starting point of AD pathology lies outside the brain, and could be local or systemic inflammation due to a microbial infection.

There are many infectious agents that have been associated with AD, including bacteria, viruses, and fungi. The presence of microbes has been correlated with cognitive decline, and some of these pathogens have been identified in postmortem brains.^54^ This suggests that infectious pathogens from the body can translocate to the brain, where our immune system responds to this. It is known the immune cells from the peripheral immune system communicate with the brains’ immune system, in which microglia play an important role.^52^

**Figure 2.**
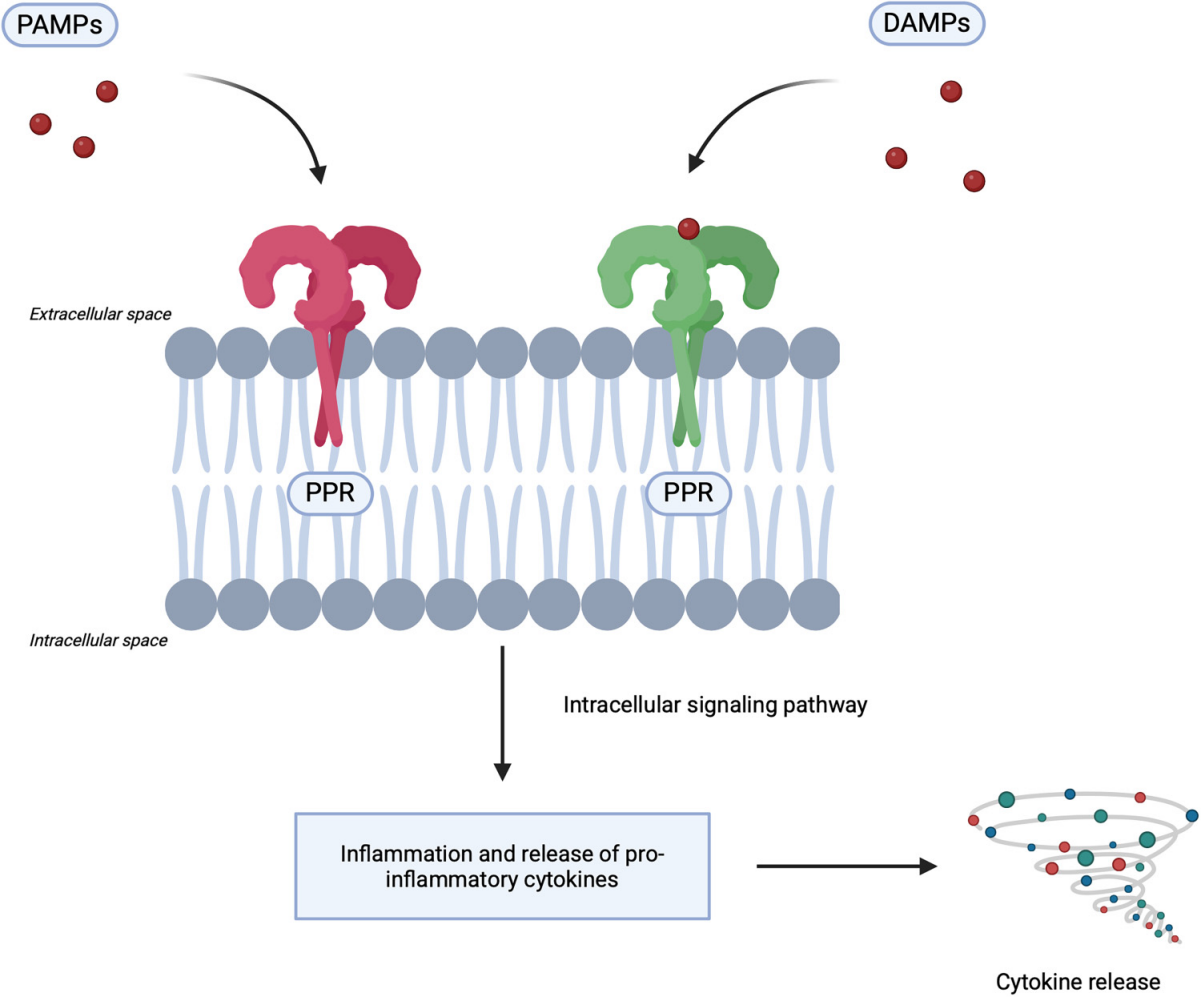
The activation process of microglia.

As mentioned previously, microglia scan their surroundings for abnormal stimuli. They express cell surface receptors, called pattern recognition receptors (PRRs), that can recognize pathogen-associated molecular patterns (PAMPs) and damage-associated molecular patterns (DAMPs). PAMPs are stimuli derived from pathogens, of which lipopolysaccharide (LPS) is an important example, and DAMPs are host-derived intracellular molecules released upon damage. This process activates microglia, resulting in the triggering of an intracellular signaling pathway, which induces the release of pro-inflammatory cytokines and inflammation. These activated microglia are called disease-associated microglia. In AD, these PRRs recognize, in addition to microbial stimuli, other pathological hallmarks, such as Aβ (Figure 2).^30,54^

Pathogen-associated molecular patterns (PAMPs). (e.g., Aβ) and damage-associated molecular patterns (DAMPs) bind to pattern recognition receptors (PRRs) on the cell surface of microglia. The activation of PRRs triggers an intracellular signaling pathway, resulting in an inflammatory response and the release of proinflammatory cytokines, including IL-1β, IL-6, and IL-8 (Created in BioRender. Evers, M. (2026) https://BioRender.com/b4sa6dg).

Furthermore, studies have shown that microglia also become activated by chronic or early-life infections. Generally, after responding to the stimulus, these microglia would return to a “resting” state, however evidence suggests that due to chronic or early-life inflammation, these microglia can remain in a “hyperactive” state allowing them to respond more sensitively to pathogens later in life, activating cell death cascades and ultimately leading to neuronal cell death.^54,55^

### The blood-brain barrier

**Figure 3.**
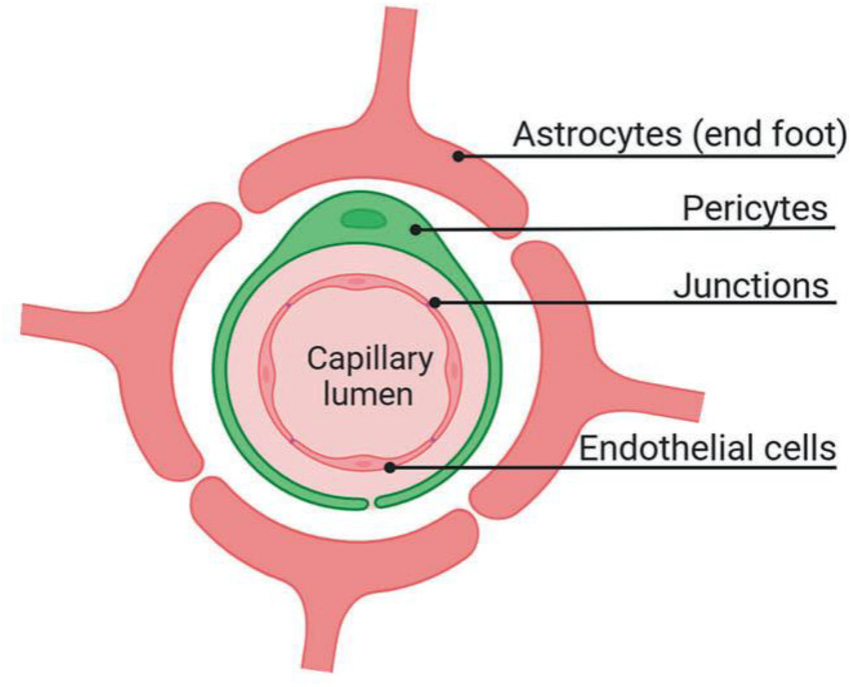
Schematic overview of the structure of the blood-brain barrier (Figure modified from Wu and colleagues).^57^

In addition to microglia, the blood-brain barrier (BBB), a structural and functional barrier that regulates the transit of molecules into and out of the brain, also plays an important role as a line of defense against infections in the brain. The BBB plays an important part in protecting the brain from microorganisms and toxic products that are circulating in our blood. It is made up from different cell types, including brain microvascular endothelial cells, astrocytes, and pericytes (Figure 3).^56^

There are three mechanisms that pathogens use to cross the BBB. First, transcellular transversal: “microbial penetration through barrier cells without any evidence of microorganisms between the cells or of intercellular tight-junction disruption”, which is used by *Escherichia coli* and *C. albicans*.^58,59^ Second, paracellular traversal: “microbial penetration between barrier cells with and/or without evidence of tight-junction disruption”. And third, the Trojan Horse mechanism: “microbial penetration of the barrier cells using transmigration within infected phagocytes”.^56^ This last mechanism is used by for instance *Bacillus anthracis*.^60^ It has been hypothesized that the Trojan Horse mechanism could be used by oral pathogens (e.g., *P. gingivalis*) to disseminate from the oral cavity into the bloodstream and migrate to the brain (reviewed by de Jongh and colleagues).^61^

Furthermore, Montagne and colleagues suggested that the BBB becomes more permeable during aging, due to a loss of cerebrovascular integrity.^62^ They quantified regional BBB permeability in human AD brain tissue by using a magnetic resonance imaging protocol. They found an age-dependent breakdown of the BBB in the hippocampus, a brain region critical for memory which is one of the first brain regions to be affected in AD. These results suggest BBB permeability plays a role in the early onset of AD, and consequently that it is possible that the aging brain is colonized by microbes that transverse this barrier. Moreover, various toxins have been associated with disruption of the BBB, including various metals, pesticides, chemicals, and gut dysbiosis.^63–68^

In conclusion, microglia and the BBB play an important role in the defense against infections in the brain. Early-life and chronic infections can cause prolonged presence of hyperactive microglia that respond more sensitively to new stimuli.^54,55^ This “hyperactive” state of microglia, induced by early-life and chronic infections, could play a vital role in neuronal cell death and ultimately in the pathogenesis of AD.

## Section II: The connection between the oral microbiome and Alzheimer's disease

While recent studies have shown there is clearly a connection between the oral microbiome and AD, causal studies are missing and the exact role the oral microbiome plays in the development of this disease is still unknown. Many microorganisms have been associated with AD, including bacteria, viruses, and fungi. Especially bacteria have been extensively investigated. Interestingly, Gram-negative bacteria are more associated with AD, as opposed to Gram-positive bacteria. This could be related to their LPS, which acts as an endotoxin that is recognized by PPRs resulting in strong inflammatory responses.^69^ Evidence suggests that byproducts of Gram-negative bacteria, including LPS and fimbriae, are able to penetrate into the brain and are associated with Aβ and tau accumulation.^70^ The following section will review oral microorganisms specifically, and elucidate the connection between AD and different oral microbes, including fungi and bacteria, particularly bacteria involved in periodontitis.

### Periodontitis

The three most common oral diseases are: dental caries, periodontal disease, and oral cancer.^71^ A previous study showed that *Streptococcus mutans*, a bacterium associated with dental caries, forms amyloid proteins *in vitro*. However, there are only scarce reports on bacteria associated with dental caries in relation to AD.^72^

Generally, there is a homeostasis between the host and bacteria in the oral cavity. Changes in the oral microbiome as a result of changes in host behavior or physiology, can disrupt homeostasis, leading to an increase in the relative abundance of species which can cause inflammation. This can result in gingivitis (i.e., bleeding gums). If not treated properly, by improving general oral hygiene, gingivitis can progress into periodontitis when bacteria bypass neutrophils and penetrate into deeper tissues. This is represented by irreversible bone resorption surrounding the tooth and can ultimately lead to tooth loss.^71,73,74^

Periodontitis is a chronic multifactorial inflammatory disease characterized by polymicrobial accumulation and dysbiosis in the oral microbiome.^75^ The composition of the oral microbiome can be influenced by factors such as diet, oral hygiene, smoking, and stress.^76^ Sudden ecological and environmental changes, together with host susceptibility, can disrupt the balance of a healthy oral microbiome and promote dysbiosis and the emergence of pathogenic bacteria that drive inflammation and periodontitis.^17,61,75^ These pathogenic bacteria have been historically categorized, after performing a clustering analysis to define bacterial communities in periodontitis, as “red complex” bacteria in the pyramid of Socranksy. These bacteria include *P. gingivalis*, *T. denticola*, and *T. forsythia*, and are strongly related to the development of severe periodontitis and tightly related to each other.^77^ Although these bacteria are broadly involved in periodontitis, recent evidence points to the role of general oral dysbiosis and the host-dependent immune response, shifting towards a multisystemic view on periodontitis.^17,75,78^ Notwithstanding, *P. gingivalis* has been extensively studied in the context of periodontitis and is often considered a keystone pathogen of the disease.^79^ *P. gingivalis* is a Gram-negative, strictly anaerobic proteolytic bacterium that produces several virulence factors that plays a role in the development of periodontitis, including LPS (general virulence factor for Gram-negative bacteria) and gingipains (specific virulence factor for *P. gingivalis*). Gingipains are cysteine proteases that degrade proteins, which serve as nutrient for *P. gingivalis*. These virulence factors are often found inside outer membrane vesicles (OMVs).^80^

Many studies have investigated the role of these virulence factors and the link between periodontitis and AD. A recent meta-analysis showed a significant relationship between periodontitis and AD [OR 1.67 (1.21–2.32)].^81^ Several studies have found periodontitis related bacteria in postmortem AD brains. Dominy and colleagues found *P. gingivalis* in postmortem AD brains. In addition, they found a significantly higher gingipain load in AD brains, compared to brains of nondemented age-matched controls. The gingipain load was significantly positively correlated with tau load.^25^ Similar results were found by Yoshida and colleagues in mice. They found hyperphosphorylated tau in *P. gingivalis* OMVs-treated mice brains.^82^ Additionally, by using quantitative polymerase chain reaction, Dominy and colleagues detected *P. gingivalis* in the CSF and in saliva samples of living patients diagnosed with AD.^25^

Poole and colleagues aimed to identify the “red complex” bacteria in AD brains. Four out of ten AD brains were *P. gingivalis-*LPS positive, and 100% of non-AD controls were negative. Interestingly, they performed immunoblot analysis and results showed that AD brains were only positive for LPS, not gingipains. *T. denticola* and *T. forsythia* were not detected, due to the antigen which was poorly recognized by the antibody, thus this experiment was not continued.^26^ Moreover, Riviere and colleagues detected several *Treponema* species in AD brains by using polymerase chain reaction, including *T. denticola, T. pectinovorum*, *T. maltophilum*, *T. medium*, and *T. socranskii*. These species are normally found in healthy oral cavities and associated with good mouth hygiene.^83^ They observed higher DNA loads for these bacteria in the cortex of AD brains, and more species were present in AD brains as opposed to brains of non-AD controls.^84^

Most convincing support for the role of oral bacteria in AD development was provided by a study by Dominy and colleagues. They conducted a mice study to investigate if oral exposure to *P. gingivalis* resulted in brain colonization and whether this correlated with Aβ plaques. Indeed, oral infection with *P. gingivalis* resulted in brain infiltration in all eight mice and increased Aβ load.^25^ Various other studies have found similar results after mice were orally infected with *P. gingivalis*.^85–87^ Moreover, Dominy and colleagues found that gingipains knockout strains showed reduced level of colonization, suggesting that gingipains play a role in the pathogenicity of *P. gingivalis*, making them targets for future interventions.^25^

Furthermore, Hu and colleagues conducted a study, in which they topically applied *P. gingivalis*-LPS into the palatal gingival sulcus in rats, which was associated with memory impairment. They found increased levels of pro-inflammatory cytokines in peripheral blood and the cortex, including IL-1β, IL-6, and IL-8. Additionally, the toll-like receptor 4/NFκB signaling pathway, an important pro-inflammatory pathway, was activated. Moreover, microglia were activated and higher levels of amyloid-β protein precursor (AβPP), Aβ, and tau were detected in the LPS group as opposed to the control group.^88^ Several other studies have found *P. gingivalis’* virulence factors to be involved in neuroinflammation and the activation of glia cells.^82,89–91^ It was found that *P. gingivalis* gingipain infected microglia promote cell migration through endosomal signaling and activation of pro-inflammatory pathways.^90,91^ Similarly, Yoshida and colleagues found OMVs in the cerebral ventricles in mice, which resulted in activation of microglia and consequently a neuroinflammatory response.^82^ Several studies have shown that *P. gingivalis* OMVs are able to disrupt tight junction proteins of endothelial cells enabling them to translocate and possibly pass through the BBB.^92–94^ Taken together, these studies support the thought that infection of the brain after traversal of the BBB by *P. gingivalis*, and the resulting immune response by microglia is involved in the development of AD.

While *P. gingivalis* is the most commonly investigated oral bacterium in its role in AD, it is unlikely to be the only associated species. *P. gingivalis* is a keystone pathogen of periodontitis, relatively easy to detect, and since there is already evidence for its presence in AD brains, it is relatively safe for researchers to continue down this path. However, this raises concerns about tunnel vision. It should not be assumed that *P. gingivalis* is the only bacterium that plays a role in AD. Recent studies possibly discovered novel bacteria that are involved in periodontitis, including *Filifactor alocis* and *Peptoanaeerobacter stomatis.*^95^ Also, novel species-species correlations have been found, and there is evidence suggesting the oral virome plays a role in periodontitis.^96,97^ This indicates that, despite the many conducted studies, there are still knowledge gaps regarding the link between periodontitis and AD, and further studies are needed to sufficiently understand the role of periodontitis in AD.

### Fungi

In addition to the possible role of bacteria in the development of AD, reports have shown that fungi also play a role in the pathogenesis of AD, of which the yeast *C. albicans* has been investigated the most. Chitin is an important structural component of cell walls in fungi specifically.^98^ A recent study found that Chitotriosidase, an enzyme that degrades chitin, is increased in cerebrospinal fluid and peripheral blood in AD patients, and that this enzyme is involved in neuroinflammatory processes and cognitive impairment.^99–101^ This suggests that fungi, possibly oral fungi specifically, play a role in the pathogenesis of AD.

It is generally accepted that *C. albicans* is the most common species of fungi present in the healthy oral cavity (reviewed by Krom et al.).^102^ Ghannoum and colleagues were the first to use next generation sequencing to investigate which fungi species are present in the healthy oral cavity. They identified 101 different fungal species. However, they reported that there was large individual variability, possibly due to environmental contamination.^103^ To this date, there is still much unknown about the fungal composition in the oral cavity and future studies should take this into consideration.

Various studies have reported the presence of fungi in AD brains and plasma. Previous studies examined postmortem AD brain tissue and found various fungal species, including multiple *Candida* species, *Cryptococcus magnum*, and *Cladosporium spp.*^104,105^ However, Alonso and colleagues found fungal DNA in controls, suggesting the presence of fungi in the central nervous system in low percentages does not have to lead to AD. They propose a certain threshold for the amount of fungal DNA and different species present in the brain. If this threshold is exceeded it can result in progressive AD symptoms.^104^ It should however be noted that DNA-based results are difficult to interpret due to the ubiquitous presence of (sporulating) fungi in our environment (e.g., *Cladosporium spp*.) leading to high risk of environmental contamination of samples.

Furthermore, several mice studies have been performed to elucidate the role of *C. albicans* in AD. *C. albicans* has two known virulence factors: candidalysin and Saps. Candidalysin is a fungal peptide toxin that is mainly known for its function to lyse epithelial cells and induce the release of inflammatory cytokines, and plays a role in mucosal infection of *C. albicans*.^106^ However, recent studies have found candidalysin to play an additional role in AD. Candidalysin activates microglia which promotes clearance from *C. albicans* from the brain.^107^ Additionally, candidalysin loaded OMVs have been identified.^108^ Furthermore, studies have shown that Saps promote the cleavage of AβPP into Aβ peptides, which promotes fungal killing. Additionally, Wu and colleagues showed that, after intravenous injection of *C. albicans* cells, microglia were activated and surrounded yeast aggregates, forming fungal-induced glial granulomas. These were associated with memory impairment. AβPP was localized within, and Aβ peptides around the fungal-induced glial granulomas. The *C. albicans* infected mice showed mild memory impairment, which returned to normal once the fungi was cleared.^109^ However, it is important to note that AD is a progressive disease and it seems unlikely that memory impairment would return to normal in humans, after clearance of microorganisms in the brain. Moreover, multiple studies have reported that Aβ has an antimicrobial nature, possibly causing an infection with *C. albicans* to result in abundant Aβ peptides, ultimately causing the formation of Aβ plaques.^110,111^

Furthermore, mouse models have shown that *C. albicans* is able to cross the BBB. Wu and colleagues found that Saps disrupted tight junctions of the BBB, which allow *C. albicans* to invade the brain.^107^ Furthermore, it was shown that *C. albicans* can penetrate the BBB and proliferate in the brain, after intravenous injection of *C. albicans*. This brain invasion resulted in short-term memory impairment.^109^ This result provides a first line of evidence for causality.

To conclude, these studies have shown that *C. albicans* is able to cross the BBB and invade the brain in mice, and the presence of various fungal species have been shown in postmortem human AD brains. These results suggest that fungi play a role in the progression of AD, and further research is needed to understand this role properly.

## Section III: A mechanistic framework describing the role of the oral microbiome in the development of Alzheimer's disease

For decades hypotheses have been proposed that aim to explain the connection between the oral microbiome and AD. Already in 1988, Mann and colleagues proposed the “Olfactory hypothesis”, which suggests that the olfactory tracts are a portal for pathogens to migrate to the brain and there contribute to the formation of plagues and tangles.^112^ Shoemark and Allen proposed that the oral anaerobes, which increase during life as a result of aging, start overgrowing and trigger an innate pro-inflammatory response. This immune response can weaken the BBB, making it possible for oral pathogens to migrate to the brain and contribute to AD pathogenesis.^113^ An alternative hypothesis was proposed describing that *P. gingivalis* is attracted to the brain, as there are many free iron molecules present in AD brains.^114^ However, to this date these hypotheses are still not able to fully explain the potential causal relationship between the oral microbiome and AD.

In the first two sections, associations between neuroinflammation and AD, as well as between oral microbes and neuroinflammation, have been discussed. Here, a mechanistic framework is presented that describes how oral microbes cause (dysregulation of) an inflammatory response resulting in molecular signatures of AD pathology in the brain. This mechanistic framework provides a foundation for future research on the question of causality.

**Figure 4.**
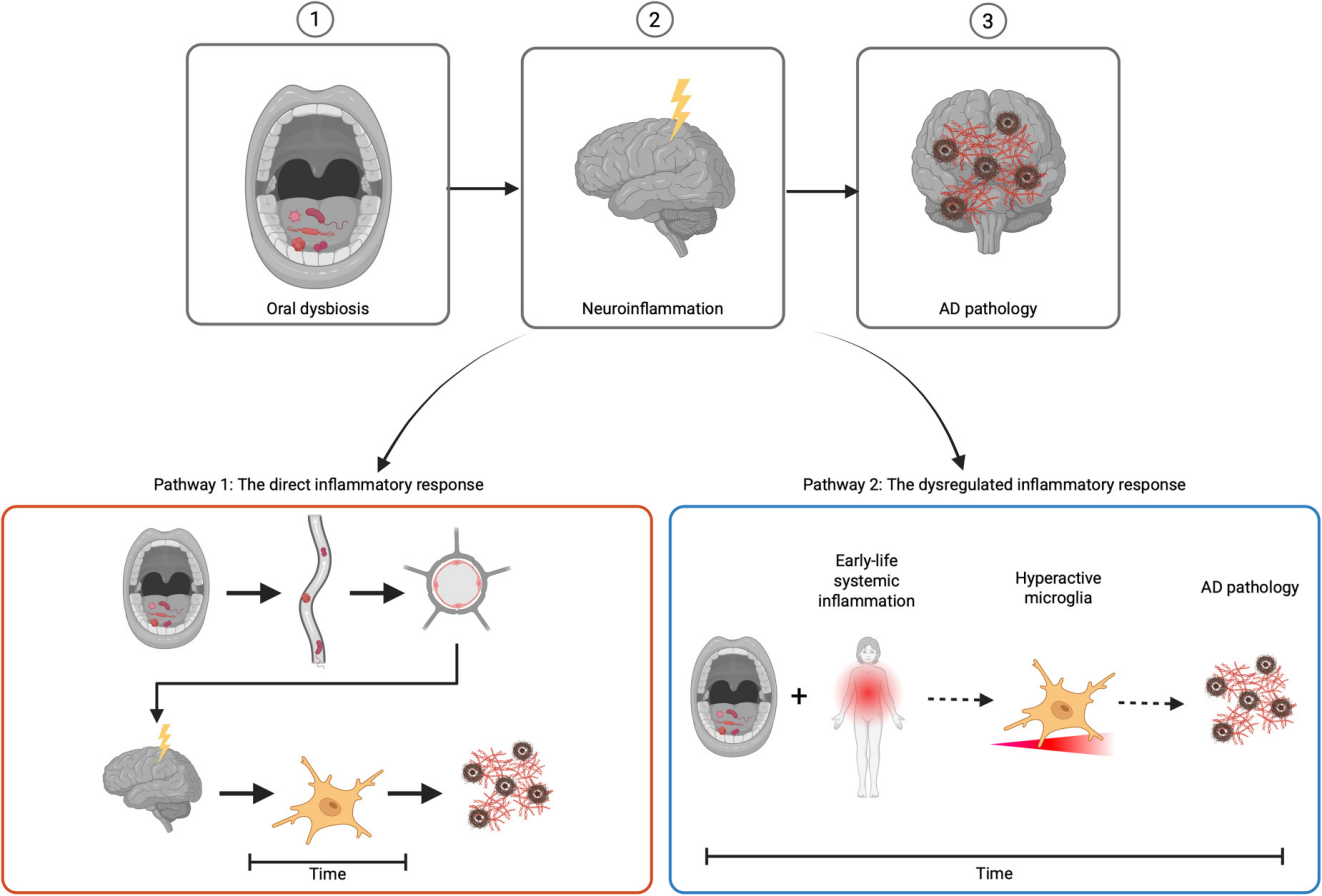
Schematic illustration of a mechanistic framework that describes the role of the oral microbiome in Alzheimer's disease. 1) Oral dysbiosis causing oral pathogens to disseminate into the bloodstream. 2) An inflammatory response is triggered which activates microglia. 3) Resulting in AD pathology. The direct inflammatory response (pathway 1) shows a direct response of the immune system in the brain to oral pathogens that migrate through the bloodstream and cross the BBB . This invasion of pathogens triggers a neuroinflammatory response, in which microglia are activated, ultimately resulting in AD pathology. This pathway occurs over a short period of time. The dysregulated inflammatory response (pathway 2) shows an early-life systemic inflammation, which causes microglia to become hyperactive, triggering many inflammatory responses over a longer period of time, ultimately resulting in AD pathology (Created in BioRender. Evers, M. (2026) https://BioRender.com/zgtixv9).

**Figure 5.**
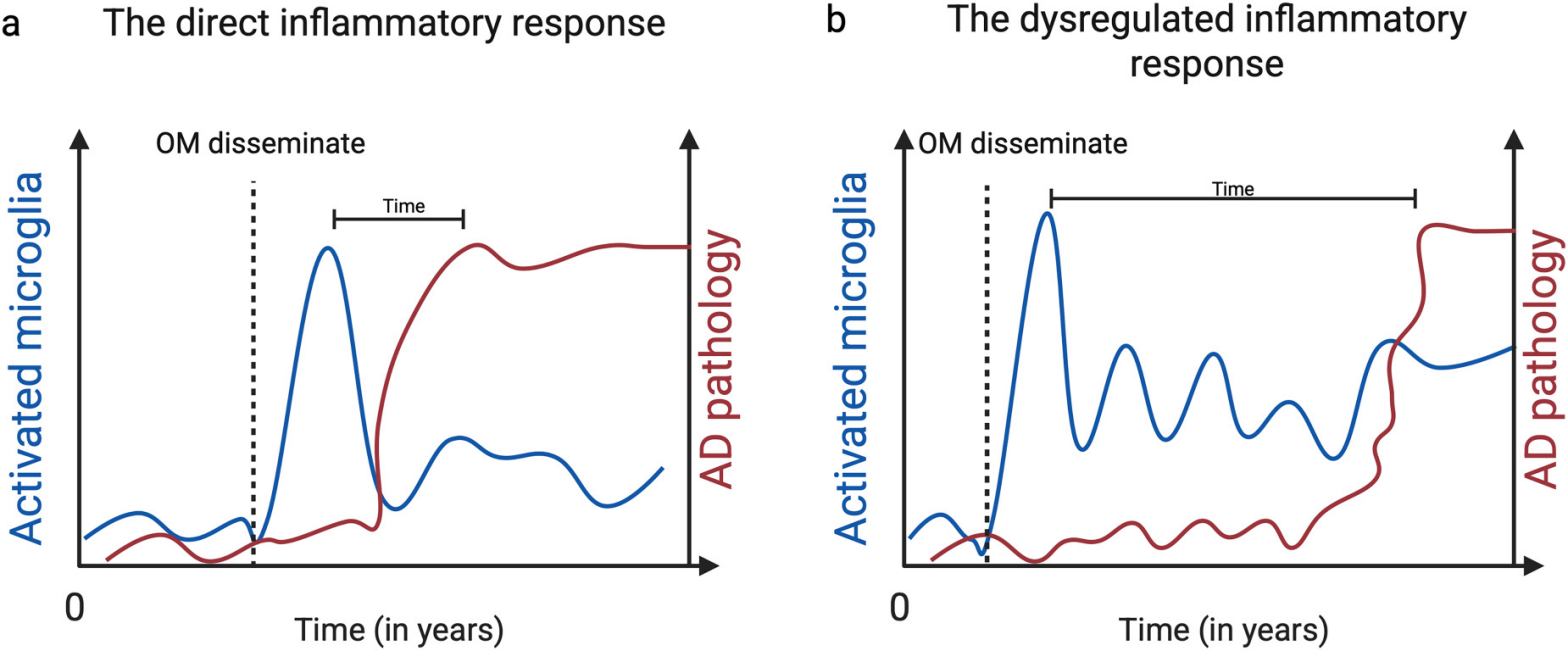
Two pathways that describe how oral microbes cause (dysregulation of) an inflammatory response resulting in molecular signatures of Alzheimer’s disease pathology. (a) The direct inflammatory response describes a direct response of the immune system in the brain on oral microbes that migrate through the bloodstream to the brain and cross the BBB. This invasion causes microglia to become activate, resulting in neuroinflammation and the formation of Aβ and tau accumulation, which ultimately results in AD pathology. (b) The dysregulated inflammatory response describes an indirect response of the immune system, on the dissemination of oral microbes and crossing the BBB during an early-life systemic inflammatory response. This results in the hyperactivation of microglia, which respond in an exaggerated way to normal stimuli, that results in inflammation and ultimately AD pathology (Created in BioRender. Evers, M. (2026) https://BioRender.com/2thytwa).

This mechanistic framework explains the role of the oral microbiome in AD through neuroinflammation and consists of a few steps described below (Figure 4). First, oral dysbiosis causing oral pathogens to disseminate into the bloodstream. This framework describes opportunistic oral pathogens, with low virulence, that cross the mucosa barrier in the oral cavity by using the mechanisms previously described (section II). Second, an inflammatory response is triggered, which activates microglia. Third, ultimately resulting in AD pathology (e.g., Aβ and tau accumulation), the pathological hallmarks of AD. From oral pathogens disseminating into the bloodstream to leading to AD pathology, two pathways can be taken. First, the direct inflammatory response: a direct response of the immune system in the brain to oral pathogens that migrate through the bloodstream and cross the BBB. This invasion of pathogens triggers a neuroinflammatory response, in which microglia are activated, ultimately resulting in AD pathology (Figures 4 and 5A). In this pathway, the presence of oral pathogens in the brain is directly linked in time to a neuroinflammatory response in which microglia play a central role. Second, the dysregulated inflammatory response: an early-life systemic inflammation causes microglia to get into a “hyperactive” state, in which they respond in an exaggerated way to normal stimuli triggering immune responses throughout a person's life that result in the accumulation of Aβ and tau (Figures 4 and 5B). This early-life systemic inflammatory response is an indirect cause of the formation of AD pathology later in life, triggered through hyperactive microglia. These pathways are not mutually exclusive and may operate in parallel or sequentially in different individuals or disease stages. The neuroinflammatory response, that forms the foundation of these pathways can be caused by oral microbes. However, it cannot be excluded that other pathogens or agents, for example microplastics or toxins, including pesticides, induces this inflammatory response, trigger hyperactive microglia at a later stage, or act in combination with oral microbes to drive inflammation. There are various studies that showed other kind of pathogens can induce AD pathology, including Herpes simplex virus, *Chlamydia pneumoniae*, and *Toxoplasma gondii*.^115–123^ Previous studies provide evidence for step one, two, and three. Studies have shown the presence of oral pathogens in postmortem AD brains, providing evidence that oral pathogens are able to disseminate into the bloodstream, cross the BBB, and invade the brain.^25,26,84^ Additionally, it has been demonstrated that microglia are activated as a response to oral pathogens invading the brain.^91,92,112^ And lastly, microglia have been linked to neuroinflammation and AD pathology.^11,12,30^ Furthermore, to substantiate the second pathway, studies have shown that microglia can get into a “hyperactive” state as a result of an early-life infection and microglial morphology shows similarities in patients who have experienced systemic inflammation and elderly with dementia.^37,54,55^ Additionally, systemic inflammation is correlated with neuroinflammation and AD, and increases the risk for development of AD.^52,53^ Generally, microglia cells return to the “resting” state after an infection, but apparently this mechanism fails at times. This raises questions, such as: “Why do in some individuals microglia do not return to a ‘resting’ state, but become hyperactive?”; “Is this dependent on the kind of infection?”; “Is there a critical period in life where infection leads to hyperactive microglia?”; “Is the ‘hyperactive’ microglia state influenced by lifestyle and/or genetics?”. Future studies should be aimed to elucidate these questions. It is known that microglia play a vital role in neuroinflammation. When these hyperactive microglia constantly “overreact” to normal stimuli over decades of time, and every time this exaggerated response causes an inflammatory response that results in neuronal damage, even though it might be a “small” inflammatory response, there could be a build-up of neuronal damage for decades, that ultimately results in AD.

Below this framework will be critically discussed. Generally, providing evidence for the cause of AD is extremely difficult. AD is a multisystemic disease and many factors may influence its pathogenesis. It seems likely that there is not just one cause for Aβ and tau accumulation. Moreover, technical limitations play a role in capturing the full complexity of the human brain. Some suggestions for future studies will be discussed below.

Previously, the study from Dominy and colleagues has been described, in which they found a significantly higher gingipain load in AD brains, compared to nondemented controls. In the additional mice study that was conducted, they found that oral infection of *P. gingivalis* resulted in brain infiltration and Aβ accumulation, however the pathogenicity of *P. gingivalis* was dependent on gingipain load.^25^ Taken this all together, this study substantiates the framework explained above. Of note, the study was performed in mice, and translation to humans is generally difficult. This translation is particularly difficult in AD studies, as mice do not develop AD naturally and mice strains are modified to guarantee the development of AD pathology. Moreover, other studies have not found gingipains in AD brains.^26^ Furthermore, Dominy and colleagues designed a small-molecule inhibitor that targets gingipains, which can be orally administered. This inhibitor, called Cortexyme, resulted in a reduction of *P. gingivalis* and reduced Aβ load, and consequently reduced neuroinflammation.^25^ Cortexyme was tested in clinical human studies, but failed in phase II/III of the clinical trial.^124^ Although failed in clinical trials, this inhibitor is a starting point and future studies could build upon this. Additionally, as previously mentioned other kind of pathogens have been shown to induce AD pathology, the most prominent example being Herpes simplex virus.^115–121^ Although, most of these studies were performed in rodents, similar studies could be designed using oral pathogens to provide further lines of evidence.

Another important note, this framework proposes that oral pathogens crossing the BBB will contribute to AD pathology. This suggests that all postmortem brains in which oral pathogens are found, would show AD pathology. However, it would seem unlikely that everyone with periodontitis, a common disease in elderly with a prevalence of 62%, which increases with age, would also develop AD.^125^ Therefore, it could be possible that a certain threshold of the amount and/or a specific combination of oral microbes or other agents that cross the BBB needs to be exceeded to acquire AD pathology. It has been shown that the BBB becomes more permeable due to aging, which makes the BBB more prone to invasion.^62^ Possibly, other barriers in the body (e.g., the mucosa barrier in the oral cavity) also weaken during aging, making it possible for oral microbes to disseminate into the bloodstream more frequent in elderly, increasing the risk of migration to the brain. The weakening of the BBB, in combination with increasing prevalence of periodontal disease in elderly, could pose increased risk of oral microbes crossing the BBB and contributing to AD pathology. This framework points to oral pathogens as an etiological agent of AD and suggest that AD prevention focusing on limiting oral pathogens from entering the bloodstream and translocating the BBB would be feasible.

Additionally, it seems likely that the immune system of the host also poses a risk factor. Cross-sectional studies suggest that diet, BMI, sleep, and especially smoking are associated with periodontitis and the prevalence seems to be higher in high-income countries.^126,127^ This would suggest that the prevalence of AD would be higher in high-income countries. It remains unclear whether this is the case, as AD is difficult to diagnose. In high-income countries only 20–50% of dementia patients receive a proper diagnosis, and in low-income countries this is only 10%.^128^ It could be argued that people in high-income countries get older and thus would be more likely to develop AD, as age is the biggest risk factor for this disease.^5^ However, epidemiological studies are thus far unable to provide evidence for that claim.

Interestingly, the prevalence of AD is higher in women, than in men.^4^ It is known that in the first trimester of pregnancy, women experience hormonal induced gingivitis. It has been hypothesized that the goal of the hormonal induced gingivitis is to allow oral microbes in the bloodstream from the pregnant mother to migrate to the placenta to “train” the immune system of the fetus.^129^ The pregnant mother experiencing gingivitis would offer increased possibility for oral microbes to migrate to the brain, which could possibly be the early-life inflammatory event as described in the dysregulated inflammatory response pathway. This would suggest that the prevalence of AD is higher in women who have been pregnant, as opposed to women who have not been. Future studies should explore the possible correlation between hormonal induced gingivitis and AD. Moreover, the permeability of the BBB could also be influenced during pregnancy. However, there are thus far no studies that can substantiate this.

To conclude, this mechanistic framework sheds new light on the mechanisms underlying the complexity of AD and provides a structured foundation for future research on the question of causality between the oral microbiome and AD and future perspectives will be discussed in the next section.

## Conclusions and future perspectives

This review explored the role of the oral microbiome in the development of AD, with microglia and neuroinflammation as potential mediators. A mechanistic framework with two pathways; the direct inflammatory response and the dysregulated inflammatory response, have been described that explain the possible causal relationship between the oral microbiome and AD. The direct inflammatory response involves microorganisms, and how low virulence oral pathogens get the opportunity to disseminate into the bloodstream and cross the BBB to cause AD. While the dysregulated inflammatory response, involves the immune system and hyperactive microglia. Here, future perspectives will be discussed that could substantiate these two pathways.

Regarding the direct inflammatory response, a longitudinal study including periodontal patients and age-matched controls would be a good starting point. This study will include patients that have an annual appointment at the dentist, and are checked on periodontitis and its progression, and simultaneously examined on Aβ and tau load by using blood biomarkers and PET scans. Moreover, statistical studies exploring the epidemiology of AD and periodontitis, the male/female prevalence, and epidemiological differences between high- and low-income countries for both AD and periodontitis are needed. Additionally, as shortly mentioned previously, there is concern about tunnel vision regarding *P. gingivalis* and its role in periodontitis and possibly AD. It is crucial for future studies to take this into consideration and take a risk on novelty by including other (oral) pathogens and investigate the role of dysbiotic communities, rather than build on previous findings only and focusing on one specific pathogen.

Furthermore, *in vitro* and *in vivo* studies exploring the “hyperactive state” of microglia are an important first step. For instance, these studies could investigate inflammatory markers associated with hyperactive microglia, DNA damage, telomere length, activity of the mTOR pathway, the production of reactive oxygen species, and other hallmarks of aging microglia.^130^ The lifespan of the cells plays an important role in the dysregulated inflammatory response, as these “hyperactive” microglia need to survive multiple years and cause inflammatory responses Although, it could also be hypothesized that the hyperactive microglia “contaminate” other microglia to become hyperactive, and it results in a cascade. Moreover, determining why some microglia return to a resting state, and others remain hyperactive is an important aspect that should be explored, by for example investigating single-cell mRNA expression, and surface expression with flow cytometry.

Additionally, investigating the permeability of various barriers is an important aspect that underlies this framework. The study from Montagne et al.^62^ is a good-starting point. Future studies could utilize *in vitro* BBB organoid models, that are cost-effective and accurate in studying the BBB and its permeability.^131^ After that, if this aging permeability process indeed poses as a risk factor for AD, future studies could explore ways to prevent this process as a therapeutic strategy.

To conclude, the mechanistic framework proposed here provides new line of thought for future studies that could contribute to a better understanding of the underlying mechanisms and pathological processes that lay at the core of the relationship between AD and the oral microbiome. It would be suggested to focus future studies on understanding these mechanisms, before investigating clinical studies, pharmacological substrates, and other therapeutic interventions.
